# Clinical characteristics and treatment of elderly onset adult-onset Still’s disease

**DOI:** 10.1038/s41598-022-10932-3

**Published:** 2022-04-26

**Authors:** Dai Kishida, Takanori Ichikawa, Ryota Takamatsu, Shun Nomura, Masayuki Matsuda, Wataru Ishii, Tatsuo Nagai, Sadahiro Suzuki, Ken-ichi Ueno, Naoki Tachibana, Yasuhiro Shimojima, Yoshiki Sekijima

**Affiliations:** 1 Department of Medicine (Neurology and Rheumatology), Shinshu University School of Medicine, 3-1-1 Asahi, Matsumoto, Japan; 2 Department of Rheumatology, Saku Central Hospital, Saku, Japan; 3 Division of Rheumatology, Department of Internal Medicine, Nagano Red Cross Hospital, Nagano, Japan; 4 Department of Rheumatology, Minaminagano Medical Center, Shinonoi General Hospital, Nagano, Japan; 5 Department of Rheumatology, Japanese Red Cross Society Suwa Hospital, Suwa, Japan; 6 Department of Nephrology, Japanese Red Cross Society Suwa Hospital, Suwa, Japan

**Keywords:** Rheumatic diseases, Inflammatory diseases

## Abstract

Adult-onset Still’s disease (AOSD)—a systemic inflammatory disease—often occurs at a young age. Recently, elderly onset patient proportion has been increasing; however, data are limited. To evaluate the characteristics of elderly patients with AOSD in a multicenter cohort, we retrospectively analyzed 62 patients with AOSD at five hospitals during April 2008–December 2020. Patients were divided into two groups according to age at disease onset: younger-onset (≤ 64 years) and elderly onset (≥ 65 years). Clinical symptoms, complications, laboratory findings, treatment, and outcomes were compared. Twenty-six (41.9%) patients developed AOSD at age ≥ 65 years. The elderly onset group had a lower frequency of sore throat (53.8% vs. 86.1%), higher frequency of pleuritis (46.2% vs. 16.7%), and higher complication rates of disseminated intravascular coagulation (30.8% vs. 8.3%) and macrophage activation syndrome (19.2% vs. 2.8%) than the younger onset group. Cytomegalovirus infections were frequent in elderly onset patients (38.5% vs. 13.9%) but decreased with early glucocorticoid dose reduction and increased immunosuppressant and tocilizumab use. Elderly AOSD is not uncommon; these patients have different characteristics than younger-onset patients. Devising a way to control disease activity quickly while managing infections may be an important goal in elderly AOSD.

## Introduction

Adult-onset Still’s disease (AOSD) is a systemic inflammatory disease characterized by spiking fever, transient rash, and polyarthritis^1,2^. Patients with AOSD have various symptoms, such as pharyngitis, lymphadenopathy, and serositis, and often develop complications, such as disseminated intravascular coagulation (DIC), macrophage activation syndrome (MAS), and amyloidosis. AOSD has aspects of an autoinflammatory disease in which innate immunity is predominant and is characterized by neutrophil and macrophage activation^3^. Increased levels of inflammatory cytokines, including interleukin (IL)-1β, IL-18, and IL-6 and increased expression of interferon-γ in natural killer cells has been reported in the acute phase of AOSD^3–5^. Both genetic and environmental factors, including various infections, are suggested to be associated with the disease onset^6^; however, the etiology of this disease remains unclear.

AOSD mainly affects young people. The age of disease onset is a bimodal peak at ages 15–25 and 36–46 years^7,8^. In Japan, the proportion of patients with elderly onset AOSD was 0–4.9% before 2000^9,10^. Two studies approximately 10 years ago revealed an elevated mean age of disease onset^11,12^, and the number of reports of patients with elderly onset AOSD has been gradually increasing^13,14^; however, information regarding the clinical features or treatment of these patients is lacking. With the aging of the population, characteristics of patients with an elderly onset have been reported in various inflammatory diseases involving innate immunity^15–17^. The number of elderly onset patients may also increase in AOSD. The treatment of AOSD have been progressed recently, and immunosuppressants, including methotrexate or cyclosporine and targeting therapy, such as IL-1 or IL-6 inhibitors, are available in addition to glucocorticoids (GCs)^18^. However, various adverse effects, including infections, easily occur in elderly patients and therefore require proper treatment. Japan has one of the highest aging rates worldwide, and this trend is even stronger in regional cities. Understanding the characteristics of patients with elderly onset AOSD is essential for appropriate management.

This study aimed to examine the clinical characteristics and treatment status of patients with elderly onset AOSD using a multicenter cohort.

## Materials and methods

### Patients

We retrospectively analyzed the clinical features and treatment of 62 adult patients with AOSD who were diagnosed between April 2008 and December 2020 at five hospitals in Nagano prefecture (Shinshu University, Saku Central Hospital, Nagano Red Cross Hospital, Shinonoi General Hospital, and Japanese Red Cross Society Suwa Hospital). AOSD was diagnosed by plural rheumatologists according to Yamaguchi’s criteria^19^. Patients diagnosed with infection, malignancy, or other rheumatic diseases were excluded. Patients were divided into two groups based on the age at disease onset, the younger-onset group (< 65 years) and the elderly onset group (≥ 65 years). Demographic data, such as age, sex, interval between onset and treatment, comorbidities, and clinical findings, such as symptoms, organ involvement, severity, complications, and laboratory data at diagnosis were examined. In addition, contents of treatments, infections during treatment, and outcomes were also examined. Regarding treatment, the number of patients received each treatment, detailed information of GCs, such as the maximum dose, the number of pulse therapy, the time until the dosage is halved, and the trends in the treatment status over time were investigated. Disease severity was evaluated using the Pouchot’s systemic score^20^. MAS was diagnosed based on the diagnostic guidelines for hemophagocytic lymphohistiocytosis-2004^21^ or the criteria for autoimmune-associated hemophagocytic syndrome^22^. Infections during treatment were defined as those requiring prolonged hospitalization or readmission. Relapse was defined as the condition of being in remission but worsened again and required intensified treatment.

This study was approved by the Institutional Review Board (No. 4529) of the Shinshu University School of Medicine, Matsumoto, Japan. All methods were carried out in accordance with relevant guidelines and regulations. Informed consent was obtained from all participants.

### Statistical analysis

Differences among categorical variables were analyzed using the Chi-squared test. Continuous variables were evaluated using Student’s *t*-test or the Mann–Whitney *U* test. Statistical significance in all analyses was set at *p* < 0.05. Values are expressed as mean ± standard deviation or median with interquartile range.

### Compliance with ethical standards

This study was performed in line with the principles of the Declaration of Helsinki. This study was approved by the Institutional Review Board (No. 4529) of the Shinshu University School of Medicine, Matsumoto, Japan. Informed consent was obtained from all the participants.

## Results

**Figure 1:**
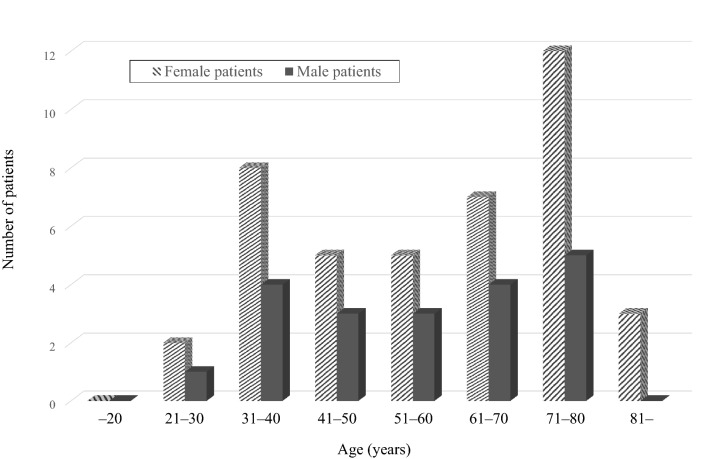
Age distribution of patients with adult-onset Still’s disease.

A total of 62 patients (42 women and 20 men) were included. The mean age at onset was 57.1 ± 17.6 years. According to the age at disease onset, 36 (58.1%) and 26 (41.9%) patients were classified into the younger-onset and elderly onset groups, respectively. The distribution of age at disease onset is shown in Fig. 1. The frequency of elderly onset AOSD was higher in female patients than in male patients (45.2% vs. 35%), although the difference was not significant.

Comparisons of the demographic, clinical, and laboratory findings between the two groups are summarized in Table 1. The male-to-female ratio and the interval between onset and treatment were not significantly different in both groups. The proportion of patients with comorbidities was higher in the elderly onset group than the younger-onset group. Comorbidities in the elderly onset group were as follows: hypertension (*n* = 8), dyslipidemia (*n* = 5), diabetes mellitus (*n* = 4), atrial fibrillation (*n* = 2), and bronchial asthma, pulmonary emphysema, angina pectoris, and osteoporosis (*n* = 1) (there was some overlapping). Meanwhile, comorbidities in the younger-onset group were as follows; hypertension (*n* = 3), and dyslipidemia, Crohn’s disease, psoriasis vulgaris, bipolar disorder, depression, and pregnancy (*n* = 1). Compared to the younger-onset group, the percentage of patients with sore throat was significantly lower, and that of pleuritis was significantly higher in the elderly onset group. In contrast, the disease severity (Pouchot’s score) and percentage of patients with fever, arthralgia, skin rash, lymphadenopathy, splenomegaly, pericarditis, interstitial pneumonitis, abdominal pain, and myalgia were not significantly different in both groups. Regarding complications, patients in the elderly onset group were significantly more likely to have DIC and MAS than those in the younger-onset group. Regarding laboratory findings, the hemoglobin level was significantly lower, and C-reactive protein level was significantly higher in patients with elderly onset AOSD than in patients with younger-onset AOSD. Although not significant, patients in the elderly onset group tended to have a higher neutrophil ratio, erythrocyte sedimentation rate, and ferritin levels than those in the younger-onset group. Positivity rates for rheumatoid factor and anti-nuclear antibody were comparable to previous studies^11,13^ and did not differ in both groups.

**Table 1:** Clinical features of patients with adult-onset Still’s disease by age group.

	Younger-onset (*n* = 36) < 65 years	Elderly onset (*n* = 26) ≥ 65 years	*p*
**Demographic features**			
Female	23 (63.9%)	19 (73.1%)	0.44
Age at onset (years)	44.5 ± 11.4	74.7 ± 5.4	** < 0.001**
Interval between onset and treatment (months)	1.0 (0.75–1.0)	1.0 (0.25–2.0)	0.67
Having any comorbidities	8 (22.2%)	12 (46.2%)	**0.046**
**Clinical symptoms**			
Fever (≥ 39 °C, lasting 1 week or longer)	36 (100%)	26 (100%)	–
Arthralgia (lasting 2 weeks or longer)	33 (91.7%)	23 (88.5%)	0.67
Skin rash	29 (80.6%)	23 (88.5%)	0.40
Typical skin rash	21 (58.3%)	14 (53.8%)	0.72
Sore throat	31 (86.1%)	14 (53.8%)	**0.004**
Lymphadenopathy	20 (55.6%)	13 (50.0%)	0.66
Splenomegaly	19 (52.8%)	10 (38.5%)	0.26
Pericarditis	5 (13.9%)	2 (7.7%)	0.44
Pleuritis	6 (16.7%)	12 (46.2%)	**0.011**
Interstitial pneumonitis	1 (2.8%)	2 (7.7%)	0.37
Abdominal pain	3 (8.3%)	2 (7.7%)	0.92
Myalgia	4 (11.1%)	4 (15.4%)	0.62
Pouchot’s score	5.0 (4.0–6.25)	5.0 (4.0–6.0)	0.54
**Complications**			
DIC	3 (8.3%)	8 (30.8%)	**0.022**
Macrophage activation syndrome	1 (2.8%)	5 (19.2%)	**0.03**
Amyloidosis	0 (0%)	0 (0%)	-
**Laboratory findings**			
White blood cells (/mm^3^)	13,125 (10,115–15,990)	14,765 (10,852–20,070)	0.25
Neutrophils (%)	87.4 (82.7–89.0)	89.0 (84.0–92.6)	0.093
Hemoglobin (g/dl)	11.7 (10.9–12.8)	10.9 (9.9–12.0)	**0.031**
Platelet (× 10^3^/mm3)	22.5 (15.6–30.6)	26.6 (13.5–37.8)	0.48
ESR (mm/h)	84.0 (26.0–94.0)	88.0 (67.0–107.0)	0.10
CRP (mg/dl)	10.5 (5.8–12.9)	14.9 (9.8–21.1)	**0.021**
Ferritin (ng/ml)	7187 (1781–16,106)	15,036 (2449–25,746)	0.097
AST (IU/L)	58 (40–85)	62 (46–88)	0.39
ALT (IU/L)	45 (31–98)	40 (22–68)	0.31
Blood urea nitrogen (mg/dl)	12.2 (9.2–16.0)	13.0 (11.0–18.4)	0.18
Creatinine (mg/dl)	0.61 (0.55–0.68)	0.63 (0.48–0.82)	0.98
Triglyceride	162 (114–217)	135 (114–175)	0.37
Fibrinogen	363 (226–474)	417 (168–580)	0.39
Positive RF	4 (11.1%)	3 (11.5%)	0.95
Positive ANA	5 (13.9%)	4 (15.4%)	0.86

Data are presented as number (%), mean ± standard deviation or median (interquartile range).

*p* < 0.05, are represented in bold.

*DIC* disseminated intravascular coagulation, *ESR* erythrocyte sedimentation rate, *CRP* C-reactive protein, *AST* aspartate aminotransferase, *ALT* alanine aminotransferase, *RF* rheumatoid factor, *ANA* anti-nuclear antibody.

**Figure 2:**
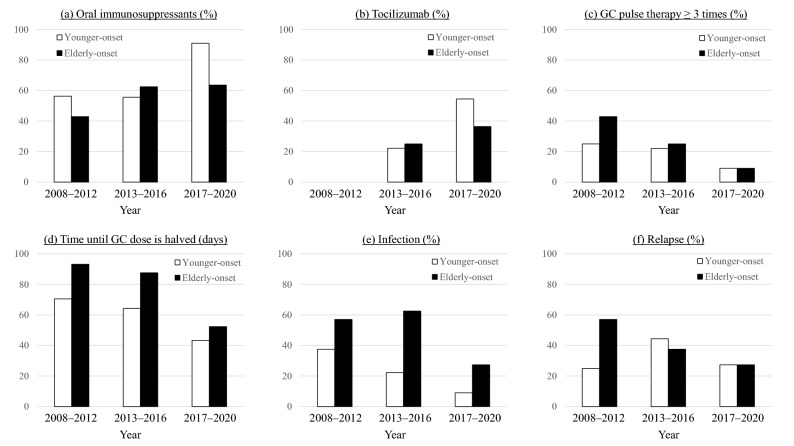
The trend of treatment status over time in patients with elderly onset and younger-onset adult-onset Still’s disease. *GCs* glucocorticoids.

The treatment and outcomes of the patients are shown in Table 2. Regarding the treatment with glucocorticoids (GCs), there were no significant differences in both groups in terms of the maximum dose, pulse therapy administration, and time until the GC dose was halved. Elderly patients had significantly more infectious episodes due to cytomegalovirus (CMV) infection than young patients. The causes of infections in the elderly onset group were Candida (*n* = 2), *Escherichia coli* (*n* = 1), and *Pseudomonas aeruginosa* (*n* = 1), whereas the cause of infections in the younger-onset group were Candida (*n* = 1), malassezia (*n* = 1), and those of unknown origin (*n* = 2). The frequencies of relapse and mortality were similar in both groups. The trends in the treatment status over time are shown in Fig. 2. The percentage of elderly onset AOSD patients using oral immunosuppressants or tocilizumab (TCZ) had increased. Meanwhile, the percentage of patients who received GC pulse therapy more than three times has decreased, and the number of days to GC halving has reduced. Moreover, the frequency of infection and relapse reduced.

**Table 2:** Treatment and outcomes of patients with adult-onset Still’s disease by age group.

	Younger-onset (*n* = 36) < 65 years	Elderly onset (*n* = 26) ≥ 65 years	*p*
**Treatment**			
GCs	35 (97.2%)	26 (100%)	0.39
Maximal dose (mg/kg/day)	0.88 ± 0.26	0.93 ± 0.29	0.47
GCs pulse therapy	26 (72.2%)	20 (76.9%)	0.67
GCs pulse therapy ≥ 3 times	7 (19.4%)	7 (26.9%)	0.48
Time until GCs dose was halved (days)	60.7 ± 25.3	72.8 ± 53.8	0.26
GCs only	11 (30.6%)	12 (46.2%)	0.20
Methotrexate	12 (33.3%)	6 (23.1%)	0.37
Tacrolimus	6 (16.7%)	2 (7.7%)	0.11
Cyclosporine	13 (36.1%)	12 (46.2%)	0.42
Tocilizumab	8 (22.2%)	7 (26.9%)	0.66
Tumor necrosis factor-α inhibitors	1 (2.8%)	1 (3.8%)	0.81
Plasma exchange	11 (30.6%)	4 (15.4%)	0.16
**Infection**			
Cytomegalovirus	5 (13.9%)	10 (38.5%)	**0.025**
Others	4 (11.1%)	4 (15.4%)	0.62
Relapse	11 (30.6%)	10 (38.5%)	0.51
Death	1 (2.8%)	2 (7.7%)	0.37

Data are presented as numbers (%) or mean ± standard deviation.

*p* < 0.05, are represented in bold.

*GC* glucocorticoid.

## Discussion

In this study, we investigated the clinical features of elderly patients with AOSD from a multicenter cohort. The results showed that elderly patients had different characteristics compared with younger patients, including less frequent sore throat, more frequent pleuritis, a higher frequency of severe complications, such as DIC and MAS, higher levels of inflammatory markers, lower levels of hemoglobin, and higher rates of CMV infections. The treatment and outcome were not significantly different in both groups; however, in the recent years, the use of immunosuppressants and TCZ instead of GCs has tended to increase, and infections and relapses have tended to decrease.

To date, some nationwide epidemiological surveys evaluating the prevalence of AOSD have been conducted in Japan using questionnaires. Before 2000, the proportion of elderly patients was very low; the first survey in 1988 showed that no patient aged ≥ 66 years developed AOSD^9^. The second survey in 1994 showed that only six patients (4.9%) were aged ≥ 65 years at the time of diagnosis^10^. The mean age at disease onset in the previous surveys in 1988 and 1994 was 32.3 and 38.1 years, respectively. A recent survey in 2010–2011 showed an elevated mean age of onset (46 years), although the proportion of patients with elderly onset AOSD was unknown^11^. An epidemiological study using the Japanese administrative database from 2010 to 2012 demonstrated that the mean age at onset was 53.1 years, and 32.1% of the patients were older than 66 years^12^. In this study, the mean age at onset was the highest (57.1 years), and the proportion of patients with elderly onset was the largest compared to previous Japanese studies. Therefore, AOSD is no longer a rare condition in old age.

Few studies have focused on the characteristics of elderly patients with AOSD. Maruyama et al.^13^ recently examined the age at disease onset in Japanese patients with AOSD and reported several similarities of patients with elderly onset AOSD with our cohort, including the frequency of sore throat, the frequency of pleuritis and DIC, and hemoglobin levels. However, there were some differences between these two studies. In our cohort, there was a high proportion of elderly patients (42% were > 65 years in our study vs. 33% were > 60 years in a previous study) and complications of MAS. Moreover, the prognosis of elderly patients in our study was similar to that of younger patients, while poor prognoses of elderly patients were shown in a previous study. Although the exact reasons for these differences remain unclear, in our study, patients with elderly onset AOSD were treated more aggressively, with a higher proportion of patients treated with methotrexate or TCZ than in previous studies. Therefore, adequate treatment, even in older patients, may lead to an improved prognosis.

In our study, elderly patients had higher levels of inflammatory markers and more frequent complications of pleuritis, DIC, and MAS than younger patients. This is not due to the delay in diagnosis because the interval between onset and treatment was not different in both groups. Macrophages play important roles in the immune response to inflammation and contribute to the onset and severity of AOSD^3^. Regarding the relationship between aging and macrophages, the nature of macrophages in the adipose tissue shifts to a pro-inflammatory type (M1) with aging^23^. Moreover, phagocytosis of apoptotic neutrophils by macrophages is weak in aging mice, making it difficult to resolve inflammation^24,25^. In addition to these immunosenescence associated with dysregulation of innate immunity, cell senescence, pro-coagulation factors, cell debris, and gut dysbiosis are causes of “inflammaging,” which is defined as chronic low-grade inflammation occurring without infection^26,27^. When some stimulation is applied to it, inflammation may persist even after the initial stimulation has been removed. Coronavirus disease 2019 is known to cause cytokine storm in some elderly patients, even though it is less common in younger patients^28^. Although it remains unknown why MAS occurs more frequently in patients with elderly onset AOSD, these inflammaging may result in more prolonged inflammation after disease onset.

CMV infections are common in elderly patients. Jia et al. reported that CMV antibodies are elevated and CMV DNA copy number is increased in patients with AOSD, suggesting that CMV may be associated with the development and exacerbation of AOSD^29^. In contrast, MAS can be triggered by various infections^30^, and there are some case reports of patients with MAS triggered by CMV infections^31,32^. In our study, MAS was frequent in elderly patients, and all cases of MAS occurred in the active state of AOSD; however, one patient had a complication of CMV infection at the development of MAS. Since CMV infections may be a risk factor for worsening AOSD and can lead to the complications of MAS, frequent monitoring and prompt treatment are important to stabilize the patient's condition.

As shown in Fig. 2, the predominant treatment for patients with elderly onset AOSD approximately 10 years ago was GCs, and more than half of them experienced some form of infection. Studies investigating the treatment of elderly patients with rheumatoid arthritis (RA) have shown that compared to younger patients, initial treatment is less aggressive, and they are less likely to receive anti-tumor necrosis factor-α therapy due to concerns of adverse events and the assumption of comorbidities^33,34^. However, in elderly patients, a delay in adequate treatment can easily lead to a decline in activities of daily living. Meanwhile, current exposure to GCs, particularly higher doses, results in the highest risk for serious infection in patients with RA^35^. Moreover, patients with elderly onset AOSD in our study had a higher frequency of comorbidities and most of these are easily deteriorated using GCs. These findings suggest that elderly patients require adequate treatment with medications other than GCs. With the elucidation of the pathogenesis, including cytokine profile of patients with AOSD, several established targeting therapies have been developed. Methotrexate and anti-cytokine therapy, such as IL-1 or IL-6 inhibitors, showed not only potent anti-inflammatory effects but also GC-sparing effects^18^. In our study, we also found that the treatment of elderly patients with AOSD changed over time. The frequency of using TCZ and immunosuppressants increased even in elderly patients, and there is a shift away from GC-dependent therapy in this group. Regarding TCZ, an available biological agent in Japan, a meta-analysis of AOSD showed excellent efficacy, especially in refractory patients, and TCZ could reduce the need for GCs^36^. Moreover, a double-blind, randomized trial of TCZ for refractory AOSD revealed significant improvement in systemic symptoms^37^. IL-1 inhibitors are also effective for patients with AOSD. Anakinra showed excellent effect for patients with predominantly systemic symptoms and revealed GC-sparing effect^38^. Efficacy and safety of canakinumab are also reported in a systematic review^39^. As IL-1 inhibitors are unavailable in Japan because of the off-label use for AOSD, none of our patients used these agents. However, it is expected that we can use several anti-cytokine therapies, including IL-1 inhibitors, in the near future. The development of methods to reduce infections must be continued through the appropriate use of drugs other than GCs, especially for elderly patients with AOSD.

This study has some limitations. First, this study has a retrospective design with a small number of patients. Second, we did not examine the information of the clinical patterns, such as monocyclic, polycyclic, and chronic articular, in this study. A nationwide survey including this information is needed in the future. Third, the rates of infection and relapse may be affected by the differences in follow-up duration; however, since most of the infectious episodes were prolonged hospitalization, the influence is likely to be limited. Fourth, the large proportion of patients with elderly onset AOSD may be because the study was conducted in Japan, the country with one of the highest aging rates worldwide. The global population is also aging; therefore, these situations may occur in every country in the future.

In conclusion, the proportion of elderly patients with AOSD gradually increases. The disease activity of patients with elderly onset AOSD was similar to that of patients with younger-onset AOSD, and the frequency of complications, such as DIC and MAS, was high. CMV infections were common in patients with elderly onset AOSD but had been decreasing in recent years with a shift away from GC-dependent treatment. In elderly patients with AOSD, the appropriate use of immunosuppressive agents, including cytokine inhibitors, may be necessary for the rapid control of disease activity and early reduction of GC dose.
